# Assessed Temperatures and Stress in Cats Using Tympanic and Rectal Thermometers

**DOI:** 10.3390/vetsci12040321

**Published:** 2025-04-01

**Authors:** Yrla Hanström, Sara Oltegen, Ida Eklund, Ellen Gröndahl, Ida Liszke, Josefin Söder

**Affiliations:** Department of Clinical Sciences, Faculty of Veterinary Medicine and Animal Science, Swedish University of Agricultural Sciences, P.O. Box 7054, 75007 Uppsala, Sweden

**Keywords:** temperature measurement, FAS, feline, fever, hypothermic, hyperthermic, in-ear temperature, normothermic, pyrexia, rectal temperature, stress

## Abstract

Previous studies using tympanic thermometers on pets have presented inconsistent conclusions. The first aim of the current study was to compare the assessed temperatures using tympanic and rectal thermometers on healthy cats in a home environment and in cats admitted to clinical care. The second aim was to compare assessed stress (Fear, Anxiety and Stress scale, FAS) in healthy cats during the tympanic and rectal measurements. The non-clinical sample included 25 cats, and the clinical sample included 36 cats. The FAS score (mean ± SD) in the non-clinical sample was significantly higher (*p* < 0.0001) during the rectal measurements (2.9 ± 0.9) than during the in-ear measurements (1.6 ± 0.9), and the temperature (mean ± SD) in both the right and left ears was higher (mean bias 0.3 ± 0.8 °C, 95% limit of agreement [−1.3, 1.8]) than the rectal temperature. The cats in the clinical sample were categorized as either hypothermic (<36.7 °C, n = 5), normothermic (36.7–38.9 °C, n = 34), or hyperthermic (>38.9 °C, n = 8) according to their rectal temperature. In the hypothermic and normothermic cats, the left ear temperature was higher (mean bias 0.4 ± 0.4 °C, [−0.4, 1.2]) than the rectal temperature. In the hyperthermic cats, the left ear temperature was slightly higher (mean bias 0.1 ± 0.3 °C, [−0.4, 0.6]) than the rectal temperature. The results indicate that the currently used tympanic thermometer can be a non-stressful tool to screen for pyrexia in cats in clinical care. However, there is a risk of normothermic cats being falsely diagnosed with pyrexia. This method is not recommended in a home environment owing to the wider limits of agreements and lower precision in the non-clinical sample, complicating the interpretation of the assessed temperatures. In the current study, there were few hypothermic (n = 5) and hyperthermic cats (n = 8), as well as cats with temperatures above 40.0 °C (n = 2); thus, further studies are needed to fully establish this method’s accuracy for these patient groups.

## Simple Summary

In a clinical examination, measuring body temperature is important for the assessment of health status. Rectal measurement is commonly performed, but the procedure is often stressful for cats and is difficult for cat owners to perform at home. An alternative to rectal measurement is in-ear measurement. The aim of the current study is to assess temperature and stress in cats using tympanic and rectal thermometers. Healthy cats in their home environment and cats with a low, normal, or high temperature admitted to clinical care were evaluated. The assessed stress level was almost twice as high during the rectal measurements compared to that in the ears. For cats at the clinic with a low or normal temperature, the tympanic thermometer showed a higher temperature compared to the rectal measurement, with a mean difference of 0.4 ± 0.4 °C. For cats with a high temperature (>38.9 °C), the mean ear temperature was 0.1 ± 0.3 °C higher than the rectal temperature. About half of the in-ear measurements of the cats with a normal temperature (36.7–38.9 °C), both at home and in clinical care, were within ±0.5 °C of the rectal temperature, but wider limits of agreements and lower precision were seen in the home environment. All the cats with a high rectal temperature were correctly classified as having a high temperature by tympanic measurement. The results indicate that taking the mean value of two tympanic measurements in the left ear can be a non-stressful method of fever screening for cats in the clinic. In cats with a low in-ear temperature (<36.7 °C), this method should be replaced by rectal measurement, as hypothermia could be more severe than what is indicated by the tympanic temperature.

## 1. Introduction

In a clinical examination, body temperature is a vital parameter for assessing the health status of cats admitted to clinical care. By measuring body temperature, the cat can be monitored for the presence of hypothermia or pyrexia. Pyrexia in cats can be caused by several underlying conditions, the most common of which are infectious diseases, inflammatory conditions, and neoplasia [1]. Stress in cats visiting a veterinary clinic may cause a slight increase in body temperature [2], and postanesthetic hyperthermia in cats may be associated with certain anesthetic agents [3]. Underlying diseases, trauma, or environmental factors may, on the other hand, cause hypothermia [4]. A low body temperature is common during or directly after the administration of anesthesia to cats [4,5]. Body temperature is a parameter that should be carefully monitored, as both severe hypothermia and hyperthermia are critical conditions in veterinary patients [3,6,7].

Rectal temperature measurement is the method most commonly used on conscious pets [8]. This method is considered reliable but has its limitations. A rectal thermometer measures the body’s core temperature by direct contact with the rectal mucosa. However, the rectal mucosa does not reflect quick temperature changes [9]. In addition, the presence of feces and conditions compromising blood flow can affect the readings [9]. The transmission of infectious diseases and the perforation of the mucosal wall in unwilling or stressed patients are also potential risks [9]. Rectal temperature measurement is often considered stressful for cats [10], not always well tolerated, and difficult for cat owners to perform at home.

Increased stress in cats may affect other vital parameters commonly monitored in clinical care [2] and contribute to the further deterioration of patients with critical conditions. An alternative to rectal temperature measurement is tympanic temperature measurement, which is commonly performed on small children [9,11,12]. The temperature regulatory center in the hypothalamus and the tympanic membrane in the ear share blood vessels and are, therefore, assumed to exhibit the same temperature [9]. A tympanic thermometer measures infrared radiation emitted from the tympanic membrane and converts radiation to a temperature reading without direct contact with the membrane [9]. Measurement is not painful, and it is fast, usually occurring within one second. There are tympanic infrared thermometers for veterinary use that have longer probes designed to fit the more complex and angled ear canals of cats and dogs [13]. Even so, measurements using veterinary tympanic thermometers have shown varied levels of accuracy when compared to the rectal measurements of cats and dogs [10,13,14,15,16,17].

In this study, a tympanic thermometer designed for people and children at least one-year-old [11] was used. Stress in cats during temperature measurement was assessed using the Fear, Anxiety, and Stress (FAS) scale [18]. The currently used tympanic thermometer has a shorter probe than the tympanic infrared thermometers deigned for veterinary use but is easily handled and available at any drug store in Sweden. It is difficult to correctly aim the probes of all tympanic thermometers toward the tympanic membrane [9]. If the temperature of the skin in the ear canal is accidentally measured, false low-temperature readings may occur [9]. Previous studies of in-ear temperature measurements using tympanic thermometers on pets have presented inconsistent conclusions. Some have found that this method might be interchangeable with rectal temperature measurement [13,16,19,20], while others have concluded the opposite [10,14,15,21]. One study used a human tympanic thermometer on healthy cats and obtained an accurate agreement of in-ear and rectal temperatures [20]. The primary aim of the current study is to compare temperature measurements using tympanic and rectal thermometers on healthy cats in a home environment and on cats admitted to clinical care with a wide temperature range. The second aim is to compare assessed stress in healthy cats during the tympanic and rectal measurements.

## 2. Materials and Methods

### 2.1. General Study Design

This study evaluated two samples of temperature measurements in cats. One study sample included healthy cats in their home environment, and the other study sample comprised cats visiting veterinary clinics. In both study samples, demographic data on the cats were obtained in addition to the temperature data. Both study samples were non-probability samples (i.e., convenience samples) based on voluntary participation. Cats showing excessive stress during the study procedures were excluded. Temperature measurements were performed primarily by four veterinary nursing students in the last semester of their Bachelor Veterinary Nursing Program at the Swedish University of Agricultural Sciences, Uppsala. Both study samples received ethical approval (Dnr 5.8.18-15533/2018) by the Ethics Committee for Animal Experiments, Uppsala, Sweden. Informed written consents from the cat owners were obtained before the inclusion of cats in the study.

#### 2.1.1. Thermometers

The tympanic thermometer used was a Braun ThermoScan 7 IRT 6520 (Kaz Europe Sàrl, Lausanne, Switzerland) with a Braun ThermoScan Hygiene Cap (Kaz Europe Sàrl, Lausanne, Switzerland) on top. The rectal thermometer used was a Braun High-Speed Thermometer PRT1000 (Kaz Europe Sàrl, Lausanne, Switzerland) with a Tempasept thermometer cover (Minitube AB, Trångviken, Sweden) and lubricant (ACO Hud Nordic AB, Upplands väsby, Sweden) on top.

#### 2.1.2. Performance of Temperature Measurements

Temperature data were collected at three anatomical locations: rectum, left ear, and right ear. For temperature measurements with the tympanic thermometer, the outer ear of the cat was gently pulled upward, and the probe was inserted as deeply as possible into the ear canal, with the probe aiming in a medio-rostral direction toward the tympanic membrane. The same hygiene cap was used for measurements of replicates in the same ear, but the cap was renewed between ears. For temperature measurements with the rectal thermometer, the tip was inserted 1–2.5 cm into the rectum. For both procedures, one person performed the temperature measurement while another person gently restrained the cat.

### 2.2. Study Population

#### 2.2.1. Non-Clinical Sample: Comparisons of Rectal, Left, and Right Ear Temperatures and Assessed Stress in Healthy Cats in Their Home Environment

A non-clinical study was conducted to investigate the comparability between temperatures assessed by a tympanic thermometer designed for humans and a rectal thermometer in healthy cats in their home environment. The study also assessed induced stress during temperature measurements with the two methods according to the Fear, Anxiety, and Stress (FAS) scale [18]. Only the FAS scale was used. The cats were recruited from Uppsala and its surroundings through social media and by private contacts. The cats were considered healthy by their owners and had to be older than one year.

The cats were randomized into two groups of equal size, where one group started with the rectal temperature measurements, and the other group started with the ear temperature measurements. Temperature measurements with the tympanic thermometer always began with the left ear, followed by the right. The rationale for this was based on the fact that the temperature in the right ear of cats might be slightly higher than the left [13] and/or more affected by stress [22]. We wanted to assess the temperature of both ears but concluded that we were more interested in the left ear temperature and, therefore, performed that measurement first. One measurement was performed in the rectum and 1–3 replicates were performed in each ear, depending on cat allowance. Two female nursing students performed all of the temperature measurements. The cats were divided so that the nurses measured half of each sample group in a randomized starting order. All measurements were performed successively on the same cat by the same person.

The stress behavior of the cats during the measurements was assessed according to the FAS scale [18]. The scale ranges from 0 to 5, where different behaviors in the animal adhere to different levels of “fear, anxiety, and stress” (0—no signs of FAS and 5—severe signs of FAS, including aggression). If a cat exhibited an FAS level of 4 or above at any time during the measurements, they were discontinued. If the cat showed an FAS level of 3, a short break was given before a new attempt was made. If the cat continued to display the same level of FAS, the measurements were discontinued.

#### 2.2.2. Clinical Sample: Comparisons of Rectal and Left Ear Temperatures in Cats Admitted to Clinical Care

A clinical study investigated the comparability of temperatures obtained by a tympanic thermometer used in the left ear and a rectal thermometer in cats admitted to veterinary care. The clinical study was supervised by two veterinary nursing students. The temperature measurements were performed by the two students as well as by other clinical staff with instruction from the students. All assessors performing temperature measurements were female.

Tympanic measurements of solely the left ear (and not of the right) were performed in the clinical sample, as the results from the left and right ear temperature measurements were comparable in the non-clinical sample. The clinical sample comprised client-owned cats admitted to three different veterinary clinics in Sweden. The veterinary clinics were of medium to large size and situated in the Stockholm area. Cats were brought to the clinics for either emergency care, pre-booked non-emergent visits to the general practice, or pre-booked surgical procedures. To be included in the study, the cats needed to tolerate temperature measurements without showing signs of excessive stress, comparable to a maximum FAS level of 4, but the FAS score was not recorded as data in this study sample.

The two veterinary nursing students, as well as seven employees at the veterinary clinics, performed the temperature measurement. Prior to the study, the clinic staff received instructions on how to properly handle the tympanic thermometer for temperature measurements. For each cat, the rectal temperature was first measured in one replicate, and the left ear was then measured in two replicates with the tympanic thermometer, as the results from the non-clinical sample indicated that beginning with rectal measurements did not affect the left ear temperature. Owing to reasons unrelated to the current study, four cats had their tympanic temperature measured first, followed by measurement of the rectal temperature. In all cats, a maximum of 15 min was allowed to pass between the rectal and in-ear measurements to avoid possible changes in body temperature that could affect the interpretation of the study results. The cats booked for surgical procedures had their temperature measured within 20 min after the administration of pre-operative sedation and/or directly after the completion of the surgical procedure. A few cats in the clinical sample were measured multiple times and thus contributed more than one observation in the study sample.

### 2.3. Data Processing and Statistical Analyses

In this study, quantitative data were generated from temperature measurements with a rectal thermometer and a tympanic thermometer and from assessed stress (FAS). Microsoft Excel, SAS (SAS 9.4 Institute Inc., Cary, NC, USA), and GraphPad Prism (GraphPad Prism 5.0, San Diego, CA, USA) were used for data processing, statistical analyses, and creation of figures. The normal distribution of data was explored by visual assessment of residuals in the mixed-model repeated-measures analysis in SAS. Temperature and stress data in both study samples (non-clinical and clinical) were not normally distributed, and logarithmization was, therefore, applied in all the mixed models. Results are presented as mean ± standard deviation (SD) or ±standard error of mean (SEM). For calculations of precision, the mean standard error (SEM) ± SD values of the ear temperature replicates were used.

Data from the non-clinical sample were divided into two groups depending on whether the evaluation started with the rectal measurement or ear measurements. Mixed-model repeated-measures analyses were used in the non-clinical sample to assess the impact on the assessed temperatures and stress of the starting order of the methods. The mixed model compared responses between the measurement locations (rectum, left, and right ear) and between groups (start in the rectum or in ears) and as analyzed pairwise comparisons (interactions between locations and groups). Tukey–Kramer adjustments were used to correct for multiple comparisons within the mixed models. Data from the cats in the clinical sample were divided into three groups based on the rectal temperature. The cats were categorized [23] as either hypothermic (<36.7 °C), normothermic (36.7–38.9 °C), or hyperthermic (>38.9 °C) according to their measured rectal temperature.

To assess the agreement between tympanic and rectal temperatures, Bland–Altman analyses and plots were used. Bland–Altman analyses can be used to calculate the agreement between two methods (in this case, ear temperature minus rectal temperature), and the difference between the methods is plotted against the average of both methods [24]. Results are presented as mean bias ± SD and the 95% limits of agreement. Joint Bland–Altman analyses and plots were applied to the non-clinical sample with the two starting groups pooled, but for the right and left ear separately. In the clinical sample, all three temperature groups were analyzed together as well as separately. Potential proportional bias in the non-clinical sample was evaluated by visual inspection of the joint Bland–Altman plots, and Pearson correlation analyses were conducted on the difference versus the average of the two methods to analyze the relationships [25]. A joint Bland–Altman plot for all three temperature groups pooled in the clinical sample was created for the evaluation of potential proportional bias, and the difference versus the average was analyzed by Pearson correlation analysis. The threshold for statistical significance was set to *p* < 0.05 in all analyses in the study.

## 3. Results

### 3.1. Description of the Cat Population

There were 61 cats included in the study population: 25 cats in the non-clinical sample and 36 cats in the clinical sample (Table 1). One cat was excluded from all measurements (in the non-clinical sample) and is not included in Table 1. The mean ages in both study samples were similar, around 4.5 years. The most common breed in both study samples was the domestic house cat, making up two-thirds of the total population. There was an almost equal division of male and female cats in both study samples (Table 1). Body weight and neutering status were not recorded in the non-clinical sample. In the clinical sample, all cats, except one male and one female cat, were neutered.

### 3.2. Non-Clinical Sample

#### 3.2.1. Descriptive Statistics of Temperature Data and Precision of Ear Temperature Measurements

Seven cats were excluded from the rectal measurements, one from measurement in the left ear, and four from measurement in the right ear because they met the cut-off level for stress. Data from two rectal measurements and data from one right ear measurement were excluded because of technical issues. A total of 58 measurements were performed in the left ear, 50 in the right ear, and 17 rectally (Supplementary File S1, Raw data). Mean temperatures in the rectum versus those in the left and right ear are shown in Table 2. The precision levels (mean SEM) of left and right ear measurements were almost similar between groups, except for the cats starting with rectal measurements, which showed a numerically higher precision level (lower mean SEM) of the right ear (Table 2).

#### 3.2.2. Comparisons of Rectal, Left, and Right Ear Temperatures

**Figure 1:**
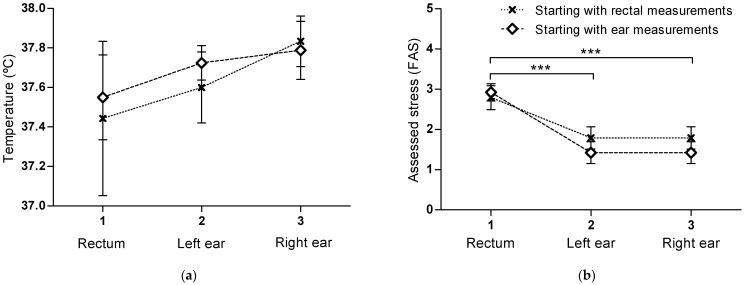
Temperature (**a**) and assessed stress (FAS) (**b**). Mean values for the different locations (rectum, left, and right ear) are marked with a star (cats starting with rectal measurements) and a rectangle (cats starting with ear measurements) (**a**,**b**). The bars represent SEM. The assessed temperatures were not affected by the starting order of the two methods (*p* = 0.10) (**a**). The assessed stress (FAS) was not affected by the starting order (*p* = 0.16), and the FAS was significantly higher when cats were measured rectally than in the ears (*** *p* < 0.001) (**b**). Number of cats, n = 25.

**Figure 2:**
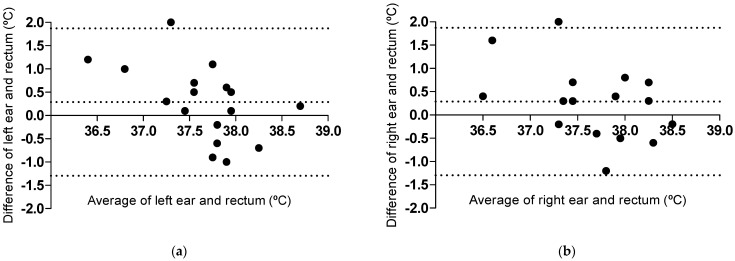
Bland–Altman plots of left ear and rectal temperatures (**a**) and right ear and rectal temperatures (**b**). Temperatures were assessed by a tympanic thermometer and a rectal thermometer. The difference between ear minus rectal temperatures (y-axis) and the average of ear and rectal temperatures (x-axis) are shown. The central dotted line represents the mean bias, and the peripheral dotted lines represent the upper and lower 95% limits of agreement. Cats with rectal, left, and right ear measurements are included, n = 17. Cats from the two starting groups were pooled in these joint plots, as the starting order of the two methods did not affect assessed temperatures.

The assessed temperatures were not affected by the starting order of the two methods (*p* = 0.10) (Figure 1a). Because starting with rectal or in-ear measurements did not affect the assessed temperatures, temperature measurements from both starting groups were pooled in joint Bland–Altman analyses and plots (Figure 2), and cats with rectal, left, and right ear measurements (n = 17) were included in separate plots per ear. The assessed temperature in both the right and left ear was higher (mean bias 0.3 ± 0.8 °C, 95% limits of agreement [−1.3, 1.8]) than the rectal temperature (Figure 2). Visual inspection of proportional bias indicated a negative linear relationship between the difference and the average with a significant correlation (*p* = 0.03, R^2^ 0.27) in the left ear (Figure 2a). The right ear (Figure 2b) showed the same tendency, but the correlation was not significant (*p* = 0.08, R^2^ 0.19). Both ears indicated smaller differences between the two methods as the temperature increased (Figure 2). A total of 7 of 17 ear temperature measurements in the left ear and 10 of 17 in the right ear were within ±0.5 °C, compared to the rectal temperature, i.e., in a total 17/34 measurements (50%).

#### 3.2.3. Comparisons of Assessed Stress Levels During Rectal and Ear Temperature Measurements

As a whole, the mixed model showed a significant difference in assessed stress (FAS) levels during temperature measurements in the rectum, left, and right ear (*p* < 0.001). The assessed mean FAS score was significantly higher (*p* < 0.0001) during the rectal measurements (2.9 ± 0.9) than during the left and right ear measurements (1.6 ± 0.9) (Figure 1b). The assessed stress was thus almost twice as high when cats were measured rectally compared to in the ears. The assessed FAS score did not differ between the left and right ear measurements (*p* = 0.95). The level of assessed stress was not affected by the order of performing the two methods (*p* = 0.16).

### 3.3. Clinical Sample

#### 3.3.1. Descriptive Statistics of Temperature Data and Precision of Ear Temperature Measurements

No cats were excluded from any measurements. Of the 36 cats included in the clinical sample, a total of 23 cats (64%) were sedated or under anesthesia and 13 cats (36%) were awake during the temperature measurements. A total of 47 paired temperature measurements consisting of one rectal and left ear measurement in two replicates were performed on the 36 cats. The paired temperature measurements were classified as either hypothermic (n = 5), normothermic (n = 34), or hyperthermic (n = 8) according to the recorded rectal temperature (Supplementary File S1, Raw data). Mean temperatures in the rectum versus in the left ear are shown in Table 3. The precision level (mean SEM) of the left ear measurements was almost equal between the normothermic and hyperthermic cats, while hypothermic cats showed a numerically higher precision level (lower mean SEM) (Table 3).

#### 3.3.2. Comparisons of Rectal and Left Ear Temperatures

The joint Bland–Altman analysis of all temperature groups (hypothermic, normothermic, and hyperthermic) together showed a mean bias of 0.3 ± 0.4 °C, 95% limits of agreement [−0.5, 1.1]. The joint plot showed a tendency for proportional bias, but the relationship was not linear (Pearson correlation; *p* = 0.44, R^2^ 0.01) (Supplementary File S2, Figure S1). Both lower and especially higher temperatures exhibited lower differences between the two methods compared to the temperature range of normothermic cats (36.7–38.9 °C) (Supplementary File S2, Figure S1). To evaluate this aspect further, the data of the clinical sample are presented below with Bland–Altman analyses and plots per temperature group, according to the selected temperature intervals of the study [23].

**Figure 3:**
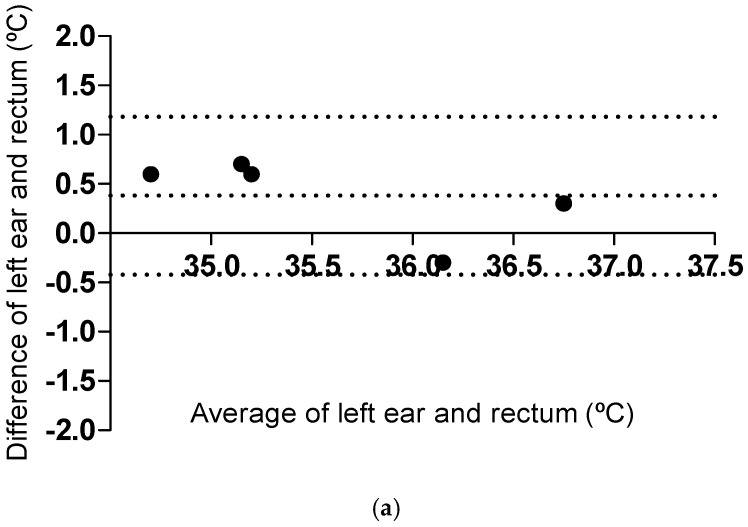

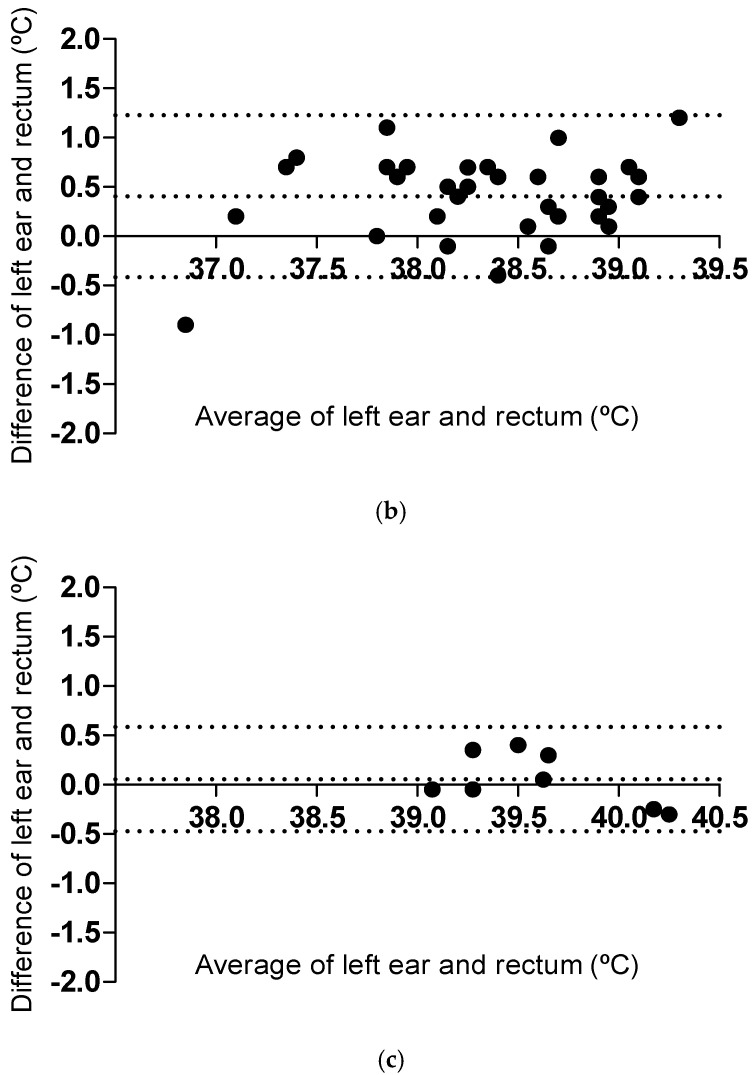
Bland–Altman plots of left ear and rectal temperatures in (**a**) hypothermic cats (<36.7 °C, n = 5), (**b**) normothermic cats (36.7–38.9 °C, n = 34), and (**c**) hyperthermic cats (>38.9 °C, n = 8). Temperatures were assessed by a tympanic thermometer and a rectal thermometer, and cats were classified according to their rectal temperature. The difference between left ear minus rectal temperatures (y-axis) and the average of left ear and rectal temperatures (x-axis) are shown. The central dotted lines represent the mean bias, and the peripheral dotted lines represent the upper and lower 95% limits of agreement.

In hypothermic and normothermic cats, the left ear temperature was higher (mean bias 0.4 ± 0.4 °C, 95% limits of agreement [−0.4, 1.2]) than the rectal temperature. In hyperthermic cats, the left ear temperature was slightly higher (mean bias 0.1 ± 0.3 °C, [−0.4, 0.6]) than the rectal temperature (Figure 3). In hypothermic cats, two of five (40%) of the temperature measurements in the left ear were within ±0.5 °C compared to the rectal temperature. In normothermic cats, 18 of 34 (53%) of the temperature measurements in the left ear were within ±0.5 °C compared to the rectal temperature. In hyperthermic cats, eight of eight (100%) of the left ear measurements were within ±0.5 °C, compared to the rectal temperature.

## 4. Discussion

This study compared temperatures assessed using a tympanic and a rectal thermometer in healthy cats in a home environment and in cats admitted to clinical care. The assessed stress was almost twice as high during the rectal measurement compared to the measurement in the ears in the healthy cats. The in-ear temperature in the home environment was higher (mean bias 0.3 ± 0.8 °C) than the rectal temperature. In hypothermic and normothermic cats at the clinic, the left ear temperature was also higher (mean bias 0.4 ± 0.4 °C) than the rectal temperature. In hyperthermic cats, the left ear temperature was slightly higher (mean bias 0.1 ± 0.3 °C) than the rectal temperature. About half of the in-ear measurements of cats with a normal temperature, both at home and in clinical care, were within ±0.5 °C compared to the rectal temperature, but wider limits of agreements and a lower precision were observed in the in-ear measurements in the home environment. In hyperthermic cats, all measurements were within ±0.5 °C. However, the numbers of hypothermic (n = 5) and hyperthermic cats (n = 8), as well as cats with temperatures above 40.0 °C (n = 2), were small, and further studies are needed to fully evaluate the method’s accuracy for these patient groups.

### 4.1. Comparisons of Assessed Rectal and In-Ear Temperatures

Three of the healthy cats in the home environment would have been classified as hypothermic by the temperature reference intervals [23] used in the clinical study sample. The in-ear temperature of these cats was higher than the rectal temperature, a result in line with the hypothermic cats at the clinic. However, the temperature difference between the methods in the hypothermic cats at home was over two times greater than in the hypothermic cats at the clinic, suggesting falsely low readings of the rectal thermometer at home. Interestingly, those three healthy cats had a low assessed stress score for the ear measurements and a high stress score for the rectal measurement, indicating that inadequate contact with the rectal mucosa might have been present due to stress behavior, such as tail tucking and movement [18]. These cats were the outliers with regard to the upper limit of agreement in the non-clinical sample (Figure 2).

Cats at a clinic are expected to experience some stress [2]. Stress in cats has been suggested to increase the temperature of predominantly the right tympanic membrane [13,22]. Interestingly, cats in the home environment that began with the more stressful rectal measurement showed numerically higher temperatures of the right ear compared to cats starting with the ear measurement, although there was no significant difference between the starting groups. If the theory of increased tympanic temperature due to increased stress [13,22] were applicable to the current study, the normothermic cats at the clinic would show a higher difference between the in-ear and rectal temperatures than the cats at home. This held true, but the numerical difference was small (mean bias 0.4 °C vs. 0.3 °C). The stress was not assessed in the cats at the clinic, so a direct comparison of stress levels between study samples was not possible. The normothermic cats in the home environment showed a considerably higher variation in their tympanic measurements compared to the normothermic cats at the clinic, indicated by a higher SD of the mean bias and wider limits of agreement. The outliers with regard to the lower limit of agreement in the non-clinical sample (Figure 2) could be falsely low readings taken by the tympanic thermometer, e.g., the temperature of the ear canal and not of the tympanic membrane [9]. This theory is supported by the mean standard error (SEM), which was about twice as high in cats at home compared to those in clinical care. A lower precision (higher SEM) of the replicates indicates that the in-ear measurements were not as repeatable at home as in the clinic, which contributed to the greater variation in the non-clinical sample.

In general, the in-ear measurements showed a higher mean temperature than was measured rectally, both in the healthy cats in the home environment and in the cats at the clinic. In-ear measurements showing higher temperatures than rectal measurements have been observed before in cats and dogs [10,20], but other studies have produced results pointing in the opposite direction, with in-ear temperatures lower than rectal temperatures [14,16,17]. In resting healthy animals in thermal homeostasis, the brain is the most metabolically active organ in the body [26] and could thus have a slightly higher temperature. Given that the rectal and in-ear temperatures assessed in the current study are correct, a higher tympanic than rectal temperature in normothermic cats could be expected from a physiological point of view. In pyrexia, the body’s metabolic rate is high in general [27]. A human study measured children with pyrexia by in-ear and rectal measurements and showed results in line with the current study, with tympanic temperatures slightly above the rectal temperatures [12]. However, in human hyperthermic patients with brain injury, the difference between brain and body temperature increased, indicating that measuring only the core temperature in brain-injured hyperthermic patients may underestimate the temperature inside the brain [26].

In a retrospective study of 275 anesthetic records of cats, 97.4% showed hypothermia during or after anesthesia [5]. In the clinical sample, two-thirds of the cats were sedated or under anesthesia during the temperature measurements, but only five cats were classified as hypothermic [23]. The tympanic thermometer assessed an in-ear temperature that was higher than the rectal temperature in the hypothermic cats. A previous study assessed in-ear temperatures in cats during general anesthesia but, in contrast to the results of the current study, found that a veterinary infrared tympanic thermometer measured a lower mean temperature compared to the rectal thermometer [14]. As hypothermia, in general, is anticipated during the anesthesia of cats [5], methods other than in-ear measurements should be used as standard procedures for temperature measurement, and more studies are needed to confirm the method’s accuracy for hypothermic cats. In addition, an unconscious cat will not be stressed by the use of a rectal thermometer.

The precision of the replicates of the in-ear measurements at home and at the clinic was generally high using the current Braun ThermoScan 7 IRT 6520. The left ear measurements in the home environment showed a mean standard error (SEM) of about 0.3 °C, and at the clinic, the mean standard error was about 0.1 °C or lower. The numerical difference in precision between the study samples could be due to cats being more confident in the home environment and, therefore, more difficult to position during the measurements; this could have compromised aiming the probe toward the tympanic membrane. Another possible explanation is that two-thirds of the measurements at the clinic were performed in anesthetized or partly anesthetized cats, which presumably facilitated an exact aiming toward the tympanic membrane. To our knowledge, no previous study has evaluated the precision (SEM) of replicates of in-ear measurements in cats, although Bland et al. (1986) described the importance of testing the repeatability of a new method [24]. A study of assessed temperatures in cats using an older version of the Braun ThermoScan showed a high correlation between in-ear measurements performed by different observers [20], indicating good inter-observer reliability, even though correlation analysis does not assure that the observers withheld the same temperature values. Rexroat et al. (1999) concluded that the variability of the in-ear measurements was slightly larger but still comparable with traditional rectal thermometers [13]. The results of the current study indicate the opposite; the variability (SD) of the mean values in both study samples showed (with one exception) numerically lower variability of the in-ear temperature measurements compared to the rectal measurements. In a study evaluating hypothermic, normothermic, and hyperthermic dogs, rectal temperature measurements showed a repeatability of mean 0.13 °C [28]. Comparing the precision of rectal measurements with the precision of in-ear measurements in the current study might have been enlightening, but the cats did not tolerate two rectal temperature measurements, and the procedure to collect rectal measurements in duplicate was discontinued at an early stage in the investigations.

### 4.2. Assessed Stress During Temperature Measurements

The assessed mean stress was almost twice as high when cats were measured rectally compared to in the ears, even though a few cats experienced the in-ear measurements as the most stressful method. Both cat owners and clinicians should, therefore, pay attention to individual preferences and, if possible, select the preferred method of the cat. Many cats did not undergo rectal temperature measurements, as the method was assessed as too stressful according to the selected cut-off. The stress level we determined during the rectal temperature measurements is, therefore, underestimated but remains almost twice as high as during the in-ear measurements. To our knowledge, no previous study has assessed stress during in-ear measurements and rectal measurements in cats with the FAS scale. A study on hospitalized dogs concluded that in-ear measurements produced a lower increase in heart rate and less defensive behavior compared to rectal measurements [16]. Smith et al. (2015) subjectively assessed how conscious cats in clinical care tolerated rectal temperature measurements. Half of the 117 cats in that study were assessed as intolerant and showed struggling, agitation, or vocalization [10], indicating a high stress level during rectal measurements. It is often anecdotally described that cats find rectal temperature measurements stressful, which is confirmed by the assessed FAS level of the current study.

### 4.3. Clinical Applications

In previous research on the use of tympanic thermometers in pets, a variety of different temperature differences have been considered acceptable compared to the rectal temperature. Two previous studies on in-ear measurements in cats set the acceptable difference at a maximum of 0.5 °C [10,20], and another study of anesthetized cats set the acceptable difference at 0.2 °C [14]. A study measuring dogs with hypothermia, normothermia, and hyperthermia found that the maximum acceptable difference between methods was 0.3 °C [21]. It is thus evident that different studies have considered different temperature differences between methods acceptable in a clinical situation. Those previous studies [10,14,20,21] evaluated temperature data that were presented as median and range, confidence intervals, mean bias and limits of agreement, complicating comparisons between studies.

In the current study, both samples showed proportional bias but of a different kind when the joint Bland–Altman plots were visually evaluated. The existence of proportional bias indicates that the methods do not agree equally through the range of measurements [25], as was illustrated by a linear relationship of the left ear in the non-clinical sample. The indication of a decreasing difference between methods as the temperature increased was further evaluated in the clinical sample. The joint Bland–Altman plot of all temperature groups in the clinical sample showed a tendency toward a proportional bias, but the relationship was not linear. Instead, both lower and especially higher temperatures indicated lower differences between methods compared to the temperature range of normothermic cats. In children, the tympanic temperature is considered to better agree with the rectal temperature in the higher temperature range, showing a good sensitivity for the determination of fever [29,30]. When proportional bias exists, regression-based 95% limits of agreement are recommended [25]. However, this was not possible in the clinical sample, as the relationship was non-linear. Therefore, the temperature data of the clinical sample were presented with separate Bland–Altman plots, one per temperature group, to enable mean bias ± SD and the 95% limits of agreement to be evaluated in hypothermic, normothermic, and hyperthermic cats, as the Bland–Altman values for the joint analysis were not completely representative of the whole temperature range of the sample.

All groups in both samples in the current study were within a temperature difference of ±0.5 °C with regard to the mean biases. However, when interpreting the mean bias, the variation (SD) of the mean bias must also be considered. The SD of the mean bias in the current study was highest in cats at home and lowest in hyperthermic cats at the clinic, and the same pattern was found in the limits of agreement. The last and perhaps the most just way to evaluate temperature data is to compare individual in-ear measurements with the rectal measurements within the same cat. In the current study, about half of the in-ear measurement of cats with a normal temperature were within ±0.5 °C compared to the rectal temperature, both at home (17 of 34) and in clinical care (18 of 34). In the hypothermic cats, only two out of five (40%) of the temperature measurements were within a difference of ±0.5 °C. In hyperthermic cats, all measurements, eight out of eight (100%), were within ±0.5 °C. Smith et al. (2015) evaluated in-ear temperatures in 150 cats in clinical care with a veterinary infrared thermometer, and their results were in line with the current study [10]. About half of their measurements were within ±0.5 °C, and the mean bias was similar even though their limits of agreement were wider [10]. In contrast to our study results, Smith et al. (2015) found that the difference between methods increased as the temperature increased [10]. Sausa et al. (2013) evaluated in-ear and rectal temperatures in 29 healthy cats daily over a 2-week period with a Braun ThermoScan IRC 4520 [20], and their results were in line with the current study. The in-ear temperature was a mean 0.1 °C higher than the rectal, in the upper range of normal for cats [23]. In addition, the majority of the tympanic measurements in that study were within ±0.5 °C of the rectal temperature. A study of dogs, similar to our results, found less difference between in-ear and rectal temperatures in patients with temperatures above 39.0 °C [16].

In normothermic cats at the clinic, the ear temperature was higher (mean 0.4 ± 0.4 °C, 95% limits of agreement [−0.4, 1.2]) than the rectal temperature, and the tympanic measurements of only 4 of 34 cats were underestimated. In hyperthermic cats, the ear temperature was only slightly higher (mean 0.1 ± 0.3 °C, [−0.4, 0.6]) than the rectal temperature, and all cats were correctly classified as “hyperthermic” by the tympanic measurements. The results thus indicate that the current tympanic thermometer, which was recommended in a recent systematic review and meta-analysis for new-generation tympanic thermometry in children [30], can be a non-stressful tool to screen for pyrexia in cats in clinical care. Nevertheless, the method is not recommended for cats in a home environment owing to its wider limits of agreements and lower precision in the non-clinical sample. There is, however, a risk of normothermic cats being falsely classified with pyrexia (>38.9 °C) as the mean bias and its SD in the normothermic group at the clinic was zero to positive (0–0.8 °C). In all, 10 of 34 cats had a temperature above 38.9 °C using the tympanic thermometer, while the same cats were below 38.9 °C according to the rectal thermometer. Two cats had a rectal temperature above 40.0 °C, which indicates more severe hyperthermia in cats [3]. Those cats were near the lower limit of agreement (Figure 3c) and, unlike the other hyperthermic cats, showed an in-ear temperature that was slightly lower than the rectal temperature. The use of the current tympanic thermometer on cats with more severe hyperthermia subsequently needs further evaluation, as an underestimation of the hyperthermic state can be fatal.

The mean bias and its SD in the hypothermic group were also zero to positive (0–0.8 °C), indicating that the hypothermia may be more severe than the tympanic temperature suggests. However, the sample of hypothermic cats was small, and more studies are needed to evaluate the method’s accuracy for this patient group [20]. If a cat with known hypothermia is approached, rectal temperature measurement should be the first method of choice. Moreover, if a cat with a hypothermic measurement is encountered (<36.7 °C), rectal measurement should replace in-ear measurement.

The current study suggests that it is difficult to altogether replace rectal temperature measurements with in-ear measurements. If using the Braun ThermoScan 7 IRT 6520 to screen for pyrexia in cats at the clinic, it is recommended to also use the Braun ThermoScan Hygiene Cap supplied by the manufacturer [11]. In the current study, the in-ear measurements were performed in duplicates of the left ear, and the device precision was good. Performing measurements in duplicate at the clinic would therefore ensure, that the temperature values obtained are alike and that the aiming toward the tympanic membrane thus is satisfactory.

### 4.4. Study Limitations and Future Perspectives

This study has several limitations. Many cats were withdrawn from rectal temperature measurements as it was assessed as too stressful for them. This resulted in only 17 rectal measurements from the cats in the home environment. However, all cats in the clinical study sample had their rectal temperatures assessed. Few hyperthermic cats were included in the study, but all showed coherent results for their left ear and rectal temperature measurements. Even fewer hypothermic cats were included, and more studies are needed to determine method accuracy for this patient group. Only two cats had a rectal temperature above 40.0 °C, and cats with more severe hyperthermia must be further investigated regarding method accuracy.

On a few occasions, a digital rectal thermometer other than that indicated was used in the clinical study sample, but this should not have influenced the results to any major extent. We would have preferred that all temperature assessments were performed by the same evaluator, but this was not possible for practical reasons. However, the precision of the tympanic thermometer remained good even though more than one evaluator performed the measurements. In addition, all evaluators were women, so the potential influence of the assessor’s gender on the level of stress was equal for all cats. The time of sampling was not known in any of the study samples, so this factor could not be investigated. The environmental temperature was not registered, but a recent study of cats showed no correlation between rectal temperature and ambient temperature [23]. The in-ear temperature could perhaps have been more influenced by the ambient temperature, but all cats were measured indoors, and thus, the environmental temperature should not have significantly influenced the results. The indications for clinical use in cats stated in this study solely adhere to the use of the current tympanic thermometer and no other models or brands, even though it is possible that other human tympanic thermometers recommended for children may perform equally well [19,20].

## 5. Conclusions

The results indicate that, based on the mean value of two replicates in the left ear, the current tympanic thermometer can be a non-stressful tool to screen for pyrexia (>38.9 °C) in cats in clinical care. However, there is a risk of normothermic cats being falsely classified with pyrexia. The method is not recommended in the home environment owing to the wider limits of agreements and lower precision in the non-clinical sample, complicating the interpretation of the assessed temperatures. In cats with a low in-ear temperature (<36.7 °C), the method should be replaced by rectal measurement, as the hypothermia could be more severe than suggested by the tympanic temperature. In the current study, there were few hypothermic (n = 5) and hyperthermic cats (n = 8) as well as cats with temperatures above 40.0 °C (n = 2), and further studies are needed to fully determine the method’s accuracy for these patient groups. The current tympanic thermometer should be further evaluated in feline patients at the clinic to verify its usability as a non-stressful method to screen for pyrexia in cats.

## Figures and Tables

**Table 1:** Descriptive statistics of the cat population in the two study samples.

	Non-Clinical Sample (n = 25)	Clinical Sample (n = 36)
Parameter	Mean ± SD	Mean ± SD
Age (years)	4.5 ± 4.2	4.5 ± 5.5
Body weight (kg)	-	3.5 ± 2.4
Breed	Number	Number
Bengal	0	2
British short hair	1	0
Devon rex	1	0
Domestic house cat	18	23
Maine coon	3	2
Neva masquerade	0	1
Norwegian forest cat	0	3
Persian	0	2
Ragdoll	2	1
Sacred Birman	0	2
Sphynx	0	1
Sex	Number	Number
Male	11	18
Female	14	18

kg: kilogram, SD: standard deviation.

**Table 2:** Mean temperatures of the starting groups for rectal and ear measurements and precision of ear measurements (mean SEM).

Group	Rectum	Left Ear		Right Ear	
Temperature (°C)	Mean ± SD	Mean ± SD	Mean SEM ± SD	Mean ± SD	Mean SEM ± SD
Starting with rectal measurements	37.4 ± 1.0	37.6 ± 0.6	0.26 ± 0.15	37.8 ± 0.4	0.16 ± 0.15
Starting with ear measurements	37.6 ± 0.7	37.7 ± 0.3	0.26 ± 0.14	37.7 ± 0.5	0.25 ± 0.24

SD: Standard deviation, SEM: Standard error of mean, Number of cats, n = 25.

**Table 3:** Mean temperatures for rectal and left ear measurements and precision of left ear measurements (mean SEM).

Group	Rectum	Left Ear	
Temperature (°C)	Mean ± SD	Mean ± SD	Mean SEM ± SD
Hypothermic	35.4 ± 1.0	35.8 ± 0.7	0.01 ± 0.02
Normothermic	38.1 ± 0.6	38.5 ± 0.7	0.10 ± 0.11
Hyperthermic	39.6 ± 0.5	39.6 ± 0.4	0.08 ± 0.08

SD: Standard deviation, SEM: standard error of mean. Cats were categorized as either hypothermic (<36.7 °C), normothermic (36.7–38.9 °C), or hyperthermic (>38.9 °C) according to their measured rectal temperature. Number of included cats: hypothermic (n = 5), normothermic (n = 34), and hyperthermic (n = 8).

## Data Availability

The data presented in this study are available in the article or in the Supplementary Materials.

## Supplementary Materials

The following supporting information can be downloaded at https://www.mdpi.com/article/10.3390/vetsci12040321/s1, Supplementary File S1: Raw data, Supplementary File S2: Figure S1.
