# Supplementary material for: Assessed Temperatures and Stress in Cats Using Tympanic and Rectal Thermometers
Source: Vet Sci. 2025 Apr 1;12(4):321. doi: 10.3390/vetsci12040321 (PMC12031553; doi:10.3390/vetsci12040321)
Supplement: Supplementary file 1 [file vetsci-12-00321-s001.zip › Supplementary File S2 -Figure S1.pdf]

Supplementary File S2, Figure S1

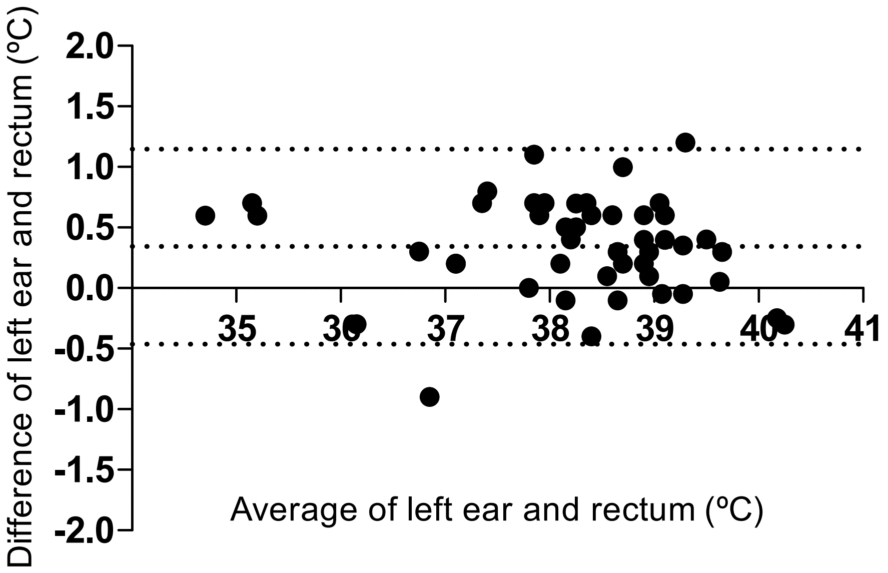

Figure S1. Joint Bland–Altman plot of left ear and rectal temperatures in hypothermic (<36.7 °C, n=5), normothermic (36.7–38.9 °C, n=34), and hyperthermic cats (>38.9 °C, n=8) in the clinical sample, plotted all together. Temperatures were assessed by a tympanic thermometer and a rectal thermometer, and cats were classified according to their rectal temperature. The difference in left ear minus rectal temperatures (y-axis) and the average of left ear and rectal temperatures (x-axis) are shown. The central dotted line represents the mean bias and the peripheral dotted lines represent upper and lower 95% limits of agreement. This joint plot of all temperature groups (hypothermic, normothermic, and hyperthermic) of the clinical sample shows a tendency toward a proportional bias, but the relationship is not linear (Pearson correlation P = 0.44, R^2 0.01). Instead, both the lower and especially the higher temperatures indicate lower differences between the two methods compared to the temperature range of normothermic cats (with a rectal temperature of 36.7–38.9 °C).
